# Factors associated with long-term clinical outcome in microscopic colitis

**DOI:** 10.1080/07853890.2024.2365989

**Published:** 2024-06-20

**Authors:** Yusuke Miyatani, Yuga Komaki, Fukiko Komaki, Dejan Micic, Kian Keyashian, Atsushi Sakuraba

**Affiliations:** a Inflammatory Bowel Disease Center, University of Chicago Medicine, Chicago, IL, USA; b Digestive and Lifestyle Diseases, Kagoshima University Graduate School of Medical and Dental Sciences, Kagoshima, Japan; c Department of Medicine, Division of Gastroenterology and Hepatology, Oregon Health & Science University, Portland, OR, USA; d Department of Medicine, Section of Gastroenterology and Hepatology, Stanford University, Stanford, CA, USA; e Division of Digestive Diseases and Nutrition, RUSH Center for Crohn’s and Colitis, RUSH University Medical Center, Chicago, IL, USA

**Keywords:** Histologic healing, microscopic colitis, long term remission

## Abstract

**Background and aims:**

Microscopic colitis has been increasingly recognized as a cause of chronic diarrhoea. We aimed to characterize the role of disease-related factors and treatments on the clinical outcomes of microscopic colitis.

**Methods:**

We retrospectively reviewed the medical records of patients with microscopic colitis who were treated at the University of Chicago and Oregon Health & Science University between August 2010 and May 2016. Patient characteristics and treatments were evaluated as predictors of clinical outcomes using univariate and multivariate analyses. Clinical remission was defined as no symptoms associated with microscopic colitis based on physician assessment and histologic remission was defined as no evidence of histological inflammation of microscopic colitis.

**Results:**

Seventy-two patients with microscopic colitis were included in the study (28 with lymphocytic colitis and 44 with collagenous colitis). Non-steroidal anti-inflammatory drugs, proton pump inhibitors and selective serotonin reuptake inhibitors were used in 23 (31.9%), 14 (19.4%) and 15 (20.8%), respectively, at the time of diagnosis. Among 46 patients with adequate follow-up data, 25 (54.3%) patients achieved clinical remission. Response to budesonide (*p* = .0002) and achieving histologic remission (*p* = .0008) were associated with clinical remission on univariate analysis. On multivariate analysis, budesonide response (*p* = .0052) was associated with clinical remission (odds ratio 25.00, 95% confidence interval 2.63–238.10). Among 22 patients who underwent a follow-up colonoscopy, five patients (22.7%) achieved histologic remission. All patients with histologic remission maintained clinical remission without medication, whereas only two patients (11.8%) were able to discontinue medical therapy when histologic inflammation was present (*p* = .0002).

**Conclusions:**

In the present cohort of patients with microscopic colitis, a favourable response to budesonide was significantly associated with long-term clinical remission, and all patients achieving histological remission were able to maintain clinical remission without further medical therapy. Larger studies are required to confirm these findings.

## 1. Introduction

Microscopic colitis is an inflammatory disease affecting the colon that causes chronic diarrhoea, mainly in older adult patients [1]. Despite a grossly normal-appearing mucosa, biopsies display histopathologic abnormalities that are subclassified into two types: collagenous colitis (CC) is characterized by thickening of the subepithelial collagen layer, and lymphocytic colitis (LC) is characterized by an increased number of intraepithelial lymphocytes [2].

The incidence of microscopic colitis has increased over the last two decades. In a North American study in 2001, the prevalence of microscopic colitis was approximately 100 cases per 100,000 persons [3]. Although this increased to approximately 220 cases per 100,000 persons in 2010, according to data from the same group, the incidence rate has plateaued in more recent years [4]. This may be attributed to the improvement in doctors’ awareness of microscopic colitis and the utilization of diagnostic methods, including colonoscopy and biopsy. However, microscopic colitis greatly affects patients’ quality of life, and the etiology and pathophysiology of microscopic colitis remain unclear.

Certain drugs, including proton pump inhibitors (PPIs) and non-steroidal anti-inflammatory drugs (NSAIDs), are associated with a risk of microscopic colitis [5,6]. While some patients’ symptoms resolve spontaneously with cessation of these drugs, most need further treatments. Budesonide has shown promising results in improving the symptoms of microscopic colitis in randomized controlled trials [7–10]. However, the long-term outcomes of microscopic colitis in real-world practice including the effects of maintenance treatment remain largely unclear [11]. Furthermore, elucidating prognostic factors for understanding long-term clinical outcomes is relevant for clinical practice.

In the present study, we aimed to investigate the clinical characteristics of patients with microscopic colitis and identify prognostic factors related to long-term clinical outcomes.

## 2. Materials and methods

### 2.1. Study design and population

Patients with microscopic colitis followed at the University of Chicago and Oregon Health & Science University between 1 August 2010 and 1 May 2016 were included, and their medical records were retrospectively reviewed. Patient characteristics at the time of diagnosis and at the first clinical visit were retrospectively evaluated. We undertook this study based on an a *priori* defined protocol, as described in the following sections. Patient demographics (age, sex, disease type and body mass index), alcohol and smoking history, concomitant and previous medication use, clinical symptoms and endoscopy and histology findings were extracted and analysed. Diagnosis of microscopic colitis was made according to published diagnostic criteria: intraepithelial lymphocytes ≥20/100 per 100 surface epithelial cells in LC and a subepithelial collagen layer thickness ≥10 µm in CC [12]. Initial medical management and changes throughout the disease course were decided by the treating physician. This study was approved by the institutional review board of each participating institution (University of Chicago: IRB16-0061 and Oregon Health & Science University: IRB00016396). Informed consent was waived due to the retrospective nature of the study.

### 2.2. Outcomes

The *priori* defined primary endpoint was clinical remission at the last visit of the study period. Clinical remission was determined based on chart documentation by the treating physician. It was determined that the patient was in clinical remission or responded to medical therapy when it was documented that the patients had stool frequency of their baseline, no urgency, no nocturnal stools, no incontinence, no weight loss and no other bowel-related symptoms. Histological remission was defined as no evidence of microscopic inflammation, as reported in the pathology report, according to the histologic diagnostic criteria [12].

### 2.3. Statistical analysis

Continuous variables are expressed as mean ± standard deviation or median and interquartile range (IQR). Differences in the mean values between subgroups were compared using the Mann–Whitney *U*-test. Categorical variables were described as percentages and compared using Fisher’s exact test. Statistical values were set at *p* < .05. Multivariate logistic regression analysis was conducted using univariate predictor variables for clinical remission with *p* values ≤.15 and odds ratios (ORs) with 95% confidence intervals (CIs) were calculated. Data were analysed using JMP software (Version 16.1.0: SAS Institute, Cary, NC).

## 3. Results

### 3.1. Patient characteristics

Patient characteristics are shown in Table 1. Our cohort included 72 patients with microscopic colitis (28 with LC and 44 with CC), with a median follow-up duration of 2 years (IQR 1.0–4.0). The mean age was 57.0 ± 16.3 years, and 61 patients (84.7%) were female, with 45 (62.5%) newly diagnosed patients. The diagnosis of microscopic colitis was based on a full colonoscopy with histopathological assessment of random biopsies obtained from the entire colon, despite no endoscopic abnormalities suggestive of other aetiologies, such as ulcerative colitis or Crohn’s disease [13]. There were 23 (31.9%), 14 (19.4%) and 15 (20.8%) patients who had a treatment history of NSAIDs, PPIs and selective serotonin reuptake inhibitors (SSRIs) at the time of diagnosis, respectively. Twenty patients (27.8%) had a history of more than one autoimmune disease, including celiac disease (*n* = 8), hypothyroidism (*n* = 9), rheumatoid arthritis (*n* = 5), psoriasis (*n* = 3) or type 1 diabetes (*n* = 1). Three patients were initially diagnosed with microscopic colitis; subsequently, their diagnosis was switched to inflammatory bowel disease (Crohn’s disease, *n* = 1; ulcerative colitis, *n* = 2) and they were not included in the subsequent analysis.

**Table 1.** Patient characteristics.

Variable	Total (*n* = 72)
Disease type	
Lymphocytic/collagenous colitis, *n* (%)	28 (38.9)/44 (61.1)
Female sex, *n* (%)	61 (84.7)
Age, mean, years (SD)	57.0 (16.3)
New diagnosis, *n* (%)	45 (62.5)
BMI, mean (SD)	24.1 (4.5)
Smoking (current/ex/non), *n* (%)	11 (15.3)/6 (8.3)/55 (76.4)
Current and ex-medication (*n*, %)	
NSAIDs	23 (31.9)
PPI	14 (19.4)
SSRI	15 (20.8)
Comorbidity of autoimmune disease, *n* (%)	20 (27.8)

### 3.2. Clinical remission

Forty-six patients underwent detailed follow-up assessments available for the assessment of clinical response. As shown in Table 2, 25 of 46 patients (54.3%) were in clinical remission at the time of the last follow-up visit. The majority of patients were treated with budesonide, and budesonide response was associated with clinical remission on univariate analysis (*p* = .0002). Achieving histologic remission (*p* = .0008) was also associated with clinical remission in univariate analysis. Multivariate analysis was conducted with factors with *p* values ≤.15 in the univariate analysis including a history of SSRI use, azathioprine or mercaptopurine use, and budesonide responders. Considering that only half of the patients had a follow-up colonoscopy with histologic assessment (*n* = 22), we excluded this from the multivariate analysis. In multivariate analysis, only budesonide responders (*p* = .0052) were associated with clinical remission, with an OR of 25.00 (95% CI 2.63–238.10). Among the 25 patients who achieved clinical remission, six had no symptoms without any maintenance medications. The majority of patients were taking low-dose budesonide as maintenance treatment.

**Table 2.** Treatment outcomes.

Variable	Clinical remission		Crude *p* value	Adjusted *p* value	Adjusted OR (95% CI)
	Yes (*n* = 25)	No (*n* = 21)			
Female sex, *n* (%)	20 (80)	18 (85.7)	.61		
Age, mean, years (SD)	59.3 (15.7)	55.6 (17.8)	.47		
BMI, mean (SD)	23.4 (3.3)	23.3 (4.6)	.99		
Diagnosis (lymphocytic), *n* (%)^a^	10 (45.5)	7 (41.2)	.79		
Alcohol consumption, *n* (%)	13 (52)	11 (52.4)	.98		
Smoking, *n* (%)	2 (8)	4 (19.0)	.27		
NSAIDs use (current or ex-), *n* (%)	11 (44)	6 (28.6)	.28		
PPI use (current or ex-), *n* (%)	5 (20)	6 (28.6)	.5		
SSRI use (current or ex-), *n* (%)	3 (12)	7 (33.3)	.081	.27	0.34 (0.050–2.50)
Statin use (current or ex-), *n* (%)	6 (24)	5 (23.8)	.41		
Budesonide use as treatment, *n* (%)	17 (68)	18 (85.7)	.16		
Budesonide responder, *n* (%)^b^	13 (61.9)	1 (5.3)	.0002	.0052	25.00 (2.63–238.10)
Mesalamine use as treatment, *n* (%)	9 (36)	8 (38.1)	.88		
Azathioprine or 6MP use as treatment, *n* (%)	1 (4)	4 (19.0)	.1	.62	0.54 (0.049–6.25)
Anti-TNFα use as treatment, *n* (%)	1 (4)	1 (4.8)	.9		
Concomitant autoimmune diseases, *n* (%)	7 (28)	8 (38.1)	.47		
Histologic remission, *n* (%)^c^	5 (62.5)	0 (0)	.0008		

^a^ Data not available in remission achieved *n* = 3, not achieved *n* = 4.

^b^ Data not available in remission achieved *n* = 4, not achieved *n* = 2.

^c^ Data available for 22 patients.

### 3.3. Association between histologic and clinical remission without maintenance medication

**Figure 1.**
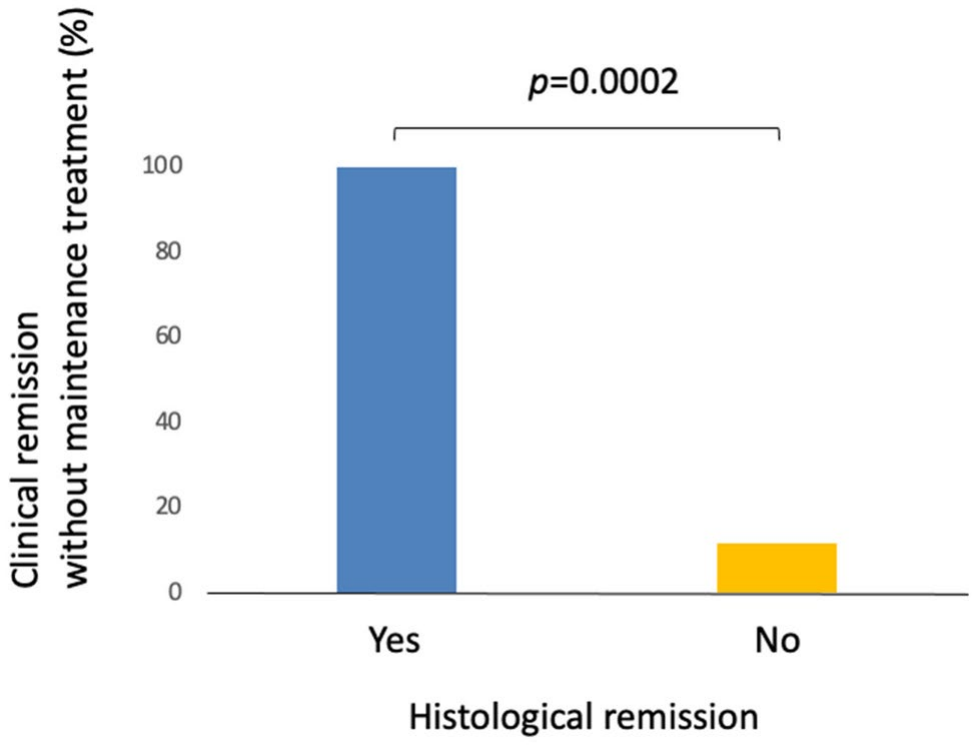
Clinical remission without maintenance treatment and histological remission. Five out of five patients (100%) who achieved histologic remission maintained clinical remission without maintenance medical treatment compared to 11.8% (two out of 17) among those with persistent histologic inflammation.

There were 22 patients who had follow-up colonoscopy with biopsy during the study period. Among them, five patients (22.7%) demonstrated a resolution of histologic inflammation associated with microscopic colitis (histologic remission). The proportion of patients who had sustained clinical remission without maintenance medication at the last follow-up visit was significantly greater among those who achieved histological remission (100%) as compared to those who had persistent histological inflammation (11.8%) (Figure 1, *p* = .0002).

## 4. Discussion

In the present study, we analysed the clinical characteristics of patients with microscopic colitis and identified the factors influencing clinical outcomes. We found that budesonide responders were significantly more likely to achieve long-term clinical remission than non-responders. Furthermore, patients with histological remission were very likely to maintain clinical remission without the need for medical therapy.

The cause of microscopic colitis is unclear, but bile acids, toxins and medications, especially NSAIDs and PPIs, play important roles in the pathogenesis of microscopic colitis [6]. These factors are thought to increase the permeability of the mucosal membrane and cause an influx of antigens into the lamina propria, resulting in inflammation. In our study, a substantial proportion of patients were taking NSAIDs, PPIs and SSRIs at the time of diagnosis, which is consistent with published literature [6]. Although those medications are risk factors for the incidence of microscopic colitis, there was no significant influence of these medications on the clinical outcome of microscopic colitis.

The majority of our patients were treated with budesonide, and achieving a response to this therapy was a prognostic factor for achieving a clinical response in univariate and multivariate analyses. Currently, budesonide is the mainstream treatment for microscopic colitis [14,15]. Previous studies demonstrated that approximately 80% of patients achieved remission with budesonide induction therapy, and more than 50% of those who responded relapsed after the cessation of budesonide during 12 months of follow-up [16]. In our cohort, only 14 (40%) patients responded to budesonide. This difference may be explained by the fact that a greater proportion of our patients had complicated disease because our hospitals were tertiary referral centres, and that patients with good responses to budesonide lacked follow-up visits.

Furthermore, a small proportion of patients achieved resolution of histological inflammation (histological remission), and these patients achieved sustained clinical remission without maintenance medication. The association between histopathological activity and clinical outcomes remains controversial. A previous study demonstrated that clinical normalization during the follow-up period is significantly associated with a decrease in intraepithelial lymphocytes [17]. On the other hand, a recent study showed no correlation between baseline histological activity and clinical symptoms, and baseline histological features did not predict the disease course at 1-year follow-up [18]. However, this study assessed histological activity at the initial diagnosis of inflammation and did not evaluate the efficacy of treatment. Our results indicate that achieving histological remission could be a target in the treatment of microscopic colitis. Further investigation is warranted to determine whether histologic remission predicts favourable long-term outcomes.

Our study has several limitations. First, our study had a retrospective design among two academic centres, so it remains to be determined whether our results are generalizable. Second, data were collected from the patients of multiple physicians; therefore, there may be interobserver variance in clinical assessment and practice patterns. Third, the sample size was not sufficient for a comprehensive multivariate analysis, and a beta error may have occurred. Lastly, data from follow-up endoscopy and histological assessment were limited. Although approximately half of the patients had follow-up colonoscopies, more patients might have achieved histological remission, since patients with improvement in their symptoms are not likely to have a follow-up colonoscopy. Prospective studies with a scheduled follow-up and colonoscopy in a larger population are warranted.

Collectively, our retrospective cohort study showed that the response to budesonide predicted long-term clinical remission, and patients achieving histologic remission were able to maintain clinical remission without medication. Larger studies are required to confirm these findings.

## Data Availability

The participants of this study did not provide written consent for their data to be shared publicly; therefore, so due to the sensitive nature of the research supporting data is not available.
